# Innovative suture technique for robotic hepaticojejunostomy: double-layer interrupted sutures

**DOI:** 10.1007/s00423-023-03020-1

**Published:** 2023-07-20

**Authors:** Kosei Takagi, Yuzo Umeda, Ryuichi Yoshida, Tomokazu Fuji, Kazuya Yasui, Takahito Yagi, Toshiyoshi Fujiwara

**Affiliations:** Department of Gastroenterological Surgery, Okayama University Graduate School of Medicine, Dentistry, and Pharmaceutical Sciences, 2-5-1 Shikata-Cho, Kita-Ku, Okayama, 700-8558 Japan

**Keywords:** Hepaticojejunostomy, Robotic surgery, Pancreatoduodenectomy, Biliary complications

## Abstract

**Purpose:**

Biliary reconstruction remains a technically demanding and complicated procedure in minimally invasive hepatopancreatobiliary surgeries. No optimal hepaticojejunostomy (HJ) technique has been demonstrated to be superior for preventing biliary complications. This study aimed to investigate the feasibility of our unique technique of posterior double-layer interrupted sutures in robotic HJ.

**Methods:**

We performed a retrospective analysis of a prospectively collected database. Forty-two patients who underwent robotic pancreatoduodenectomy using this technique between September 2020 and November 2022 at our center were reviewed. In the posterior double-layer interrupted technique, sutures were placed to bite the bile duct, posterior seromuscular layer of the jejunum, and full thickness of the jejunum.

**Results:**

The median operative time was 410 (interquartile range [IQR], 388–478) min, and the median HJ time was 30 (IQR, 28–39) min. The median bile duct diameter was 7 (IQR, 6–10) mm. Of the 42 patients, one patient (2.4%) had grade B bile leakage. During the median follow-up of 12.6 months, one patient (2.4%) with bile leakage developed anastomotic stenosis. Perioperative mortality was not observed. A surgical video showing the posterior double-layer interrupted sutures in the robotic HJ is included.

**Conclusions:**

Posterior double-layer interrupted sutures in robotic HJ provided a simple and feasible method for biliary reconstruction with a low risk of biliary complications.

**Supplementary Information:**

The online version contains supplementary material available at 10.1007/s00423-023-03020-1.

## Introduction

Although the safety and feasibility of minimally invasive pancreatoduodenectomy (MIPD) have been demonstrated [1, 2], MIPD is a technically demanding procedure that requires advanced surgical skills for performing complex reconstructions such as pancreaticojejunostomy (PJ) and hepaticojejunostomy (HJ). As biliary complications substantially impact postoperative outcomes and quality of life [3, 4], HJ is an important digestive reconstruction technique in MIPD. However, there has been an issue regarding the technical difficulties of laparoscopic HJ due to the limited movements of the needle driver [5]. In contrast, robotic surgery may overcome some of the technical challenges inherent to laparoscopic surgery [6] and enable more precise HJ anastomoses [7]. However, no optimal HJ technique has been demonstrated to be superior to other surgical techniques for preventing biliary complications [8]. Moreover, few studies have reported the surgical techniques of robotic HJ [9].

We present here the use of unique posterior double-layer interrupted sutures for HJ during robotic pancreatoduodenectomy (RPD). Furthermore, this study aimed to confirm the feasibility of this technique by investigating short-term outcomes after robotic HJ.

## Material and methods

### Study design

We performed a retrospective review of 42 consecutive patients who underwent RPD between September 2020 and November 2022 at our institution, using a prospectively collected database. Patient data included sex, age, body mass index, American Society of Anesthesiologists (ASA) physical score [10], preoperative biliary drainage (presence or absence), primary diseases, operative time, blood loss, HJ time, diameter of the bile duct, number of bile ducts (single or multiple), mortality, bile leakage, postoperative pancreatic fistula (POPF), delayed gastric emptying (DGE), and hospital stay. Bile leakage was defined and graded using the International Study Group of Liver Surgery [11]. Briefly, the severity grading of bile leakage included grade A with no change in clinical management, grade B requiring active therapeutic intervention, and grade C requiring laparotomy. The International Study Group of Pancreatic Surgery definition and grading were used to evaluate POPF and DGE [12, 13].

Regarding patient selection, the initial indication for RPD included benign and low-grade malignant tumors, but not advanced tumors [14]. The procedure was mainly performed by a single console surgeon (K.T.) who received a structured national training program for RPD in the Netherlands (LAELAPS-3) [15]. This study was approved by the Institutional Review Board of Okayama University Hospital (approval no. 2110–002).

To confirm the feasibility of our technique, we performed a literature review through PubMed using the keywords “robotic pancreatoduodenectomy,” “outcomes,” or “learning curve.” Original articles reporting the incidence of biliary complications after RPD with more than 50 cases were included.

## Surgical technique

### Robotic settings

The patient was placed in a reverse Trendelenburg position at 7° with the patient-side surgeon between the legs. The daVinci Si or Xi system (Intuitive Surgical, Sunnyvale, CA, USA) was used. An overview of surgical techniques and strategies for RPD has been previously described [14–17]. The procedure began with the extended Kocher’s maneuver, followed by dissection of the hepatoduodenal ligament, division of the pancreatic neck, and dissection of the uncinate process. HJ anastomosis was performed after PJ anastomosis using the modified Blumgart method. Finally, an antecolic gastrojejunostomy was anastomosed [17]. Two drains were placed at the PJ and HJ anastomoses, respectively.

### Robotic HJ using posterior double-layer interrupted sutures (Supplemental video)

**Fig. 1:**
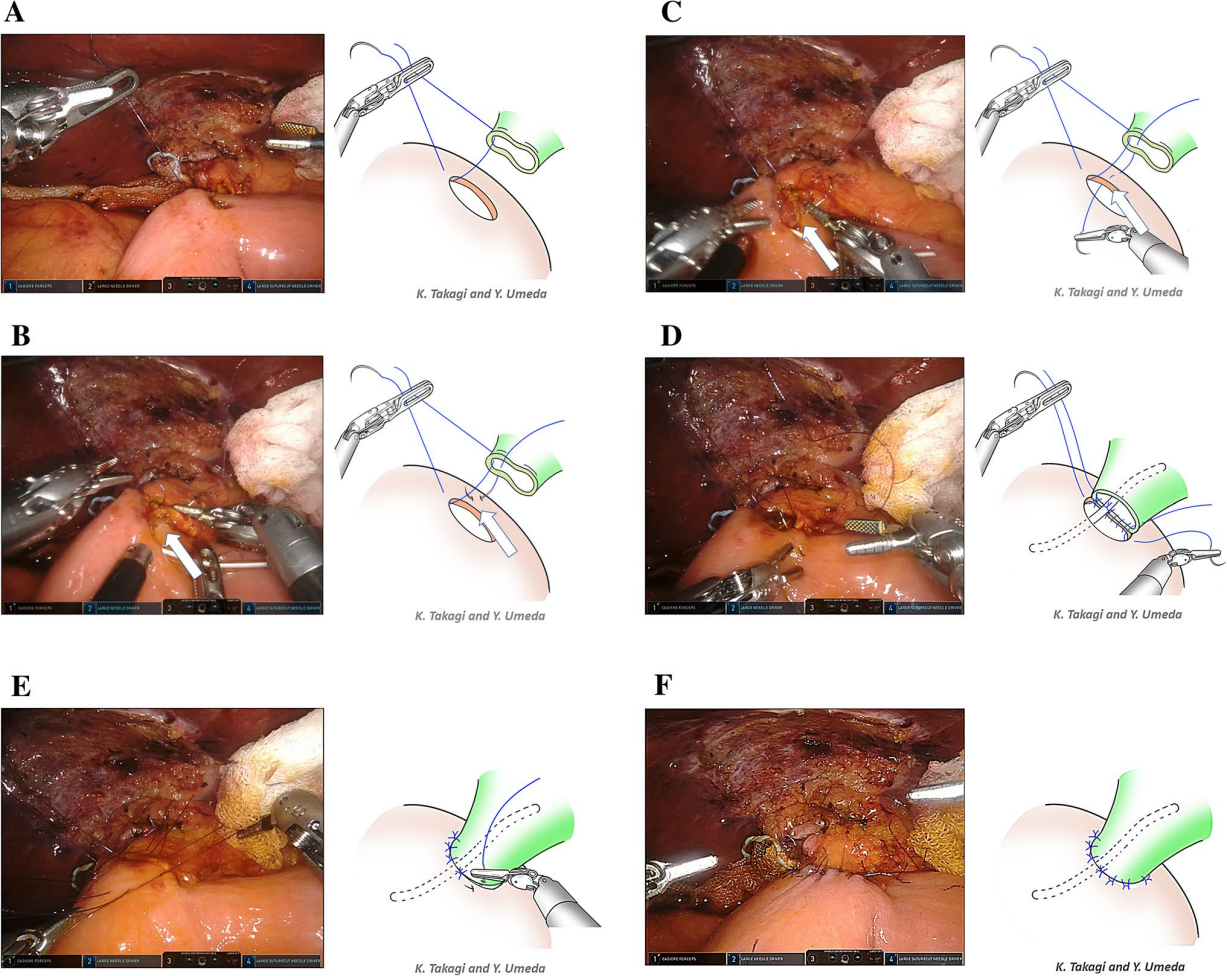
Robotic hepaticojejunostomy (HJ) using posterior double-layer interrupted sutures. **A** The first suture was established at the left corner of the bile duct (right side of the patient). The 5–0 PDS sutures were always placed from the bile duct to the jejunum. **B** Following the placement of the second suture covering the corner suture, a posterior double-layer technique was applied. The suture was placed in the bile duct and posterior seromuscular layer of the jejunum (arrow). **C** Thereafter, the full thickness of the jejunum was bitten (arrow). **D** Following completion of the posterior layer anastomosis, the intraluminal corner suture was placed on the right side of the bile duct (left side of the patient). **E** For anterior layer anastomosis, interrupted stay sutures were placed and ligated. **F** Robotic HJ anastomosis using posterior double-layer interrupted sutures was finished

For HJ anastomosis, we developed a posterior double-layer interrupted method (Fig. 1). All HJ anastomoses were performed using this technique. The large needle driver and SutureCut were used for HJ anastomosis. The 5–0 polydioxanone (PDS) sutures were always placed from the bile duct to the jejunum. The needle was turned to avoid bile duct tearing.

Initially, a small hole was made in the jejunum for anastomosis. The first suture was established at the left corner of the bile duct (right side of the patient) and retracted cranioventrally using Cadiere forceps (Fig. 1A). A second suture was placed to cover the first-corner stitch. Posterior interrupted sutures were performed using a posterior double-layer interrupted technique. The sutures were placed to bite the bile duct, the posterior seromuscular layer of the jejunum (Fig. 1B), and the full thickness of the jejunum (Fig. 1C) from the left to the right corner. Following completion of the posterior layer anastomosis, the intraluminal corner suture was placed on the right side of the bile duct (left side of the patient) (Fig. 1D). In case of a thin bile duct, a lost stent can be placed in the anastomosis. For the anterior layer anastomosis, interrupted stay sutures were placed and ligated (Fig. 1E). Finally, the robotic HJ anastomosis using posterior double-layer interrupted sutures was completed (Fig. 1F).

### Postoperative management

Oral intake was started on postoperative days (POD) 2–3. The bilirubin and amylase levels of the drain fluid were measured on POD 1, 3, and 5, as per the standard protocol. Early drain removal within 7 days after surgery was considered when no complications were suspected.

## Results

### Patient characteristics

The baseline characteristics of the 42 patients (27 men and 15 women) are shown in Table 1. Their median age was 71 (interquartile range [IQR], 56–74) years. Twenty-eight patients (67%) had ASA grade 2. Eight patients (19%) underwent preoperative endoscopic biliary drainage due to obstructive jaundice.

**Table 1:** Characteristics of the 42 patients who underwent robotic pancreatoduodenectomy

Variables	n (%) or median (IQR)
Sex	
Male	27 (64%)
Female	15 (36%)
Age, years	71 (56–74)
BMI, kg/m^2^	22.8 (21.3–24.5)
ASA score	
1	11 (26%)
2	28 (67%)
3	3 (7%)
Preoperative biliary drainage	8 (19%)
Primary diseases	
Malignant	
Ampullary carcinoma	8 (19%)
Pancreatic cancer	5 (12%)
Duodenal cancer	5 (12%)
Bile duct cancer	4 (10%)
Non-malignant	
Intraductal papillary mucinous neoplasm	7 (17%)
Duodenal tumor	6 (14%)
Others	7 (17%)

*IQR* interquartile range, *BMI* body mass index, *ASA* American Society of Anesthesiologists

### Operative outcome

The median operative time was 410 (IQR:388–478) min, including a median HJ time of 30 (IQR:28–39) min (Table 2). No conversion to open surgery was required. Regarding HJ factors, the median diameter of the bile duct was 7 (IQR, 6–10) mm. Furthermore, 31 patients (74%) had a thin bile duct of < 10 mm. Most patients had a single bile duct.

**Table 2:** Outcomes following robotic pancreatoduodenectomy

Variables	n (%) or median (IQR)
Intraoperative factors	
Operative time, min	410 (388–478)
Estimated blood loss, mL	70 (10–100)
Conversion to open	0 (0%)
HJ factors	
HJ time, min	30 (28–39)
Diameter of bile duct, mm	7 (6–10)
Diameter of bile duct < 10 mm	31 (74%)
Number of bile ducts	
Single	40 (95%)
Multiple	2 (5%)
Postoperative factors	
Mortality	0 (0%)
Bile leakage	1 (2.4%)
Grade A	0
Grade B	1
Grade C	0
POPF (grade B)	3 (7.1%)
DGE (grades B and C)	4 (9.5%)
Postoperative hospital stays, days	10 (8–14)
Follow-up after surgery, months	12.6 (9.1–19.6)

*IQR* interquartile range, *HJ* hepaticojejunostomy, *POPF* postoperative pancreatic fistula, *DGE* delayed gastric emptying

### Postoperative outcomes

Regarding postoperative short-term outcomes, one patient (2.4%) developed bile leakage that required percutaneous drainage and was evaluated as grade B. No grade C bile leakage requiring operative intervention was found. During the median follow-up of 12.6 months, the patient with bile leakage developed anastomotic stenosis, which was treated with endoscopic intervention. However, none of the other patients had bile leakage or stenosis.

### A literature review

A literature search identified eight studies reporting on the incidence of biliary complications after RPD, as demonstrated in Table 3 [15, 18–24]. The incidence of bile leakage in a multi-center study in the Netherlands (LAELAPS-3) was 10.9% [15]. The average rate of biliary complications after RPD was 6.8% (range 2.0–11%). Regarding HJ techniques, a single-layer end-to-side HJ with continuous or interrupted sutures was standard procedure during RPD.

**Table 3:** List of literatures reporting on biliary complications after following robotic pancreatoduodenectomy

Study	Year	Country, type	No. of cases	Study period	Biliary complications	Techniques for HJ
Zwart et al. [15] (LAELAPS-3)	2021	The Netherlands, multi-center	275	2016–2019	30 (10.9%)	A single-layer, end-to-side HJ
Chao et al. [18]	2023	Taiwan, single center	75	2015–2021	5 (6.7%)	A single-layer, continuous end-to-side HJ
Shi et al. [19]	2020	China, single center	200	2017–2018	11 (5.5)	A single-layer, continuous or interrupted end-to-side HJ
Zhang et al. [20]	2018	China, single center	100	2012–2016	11 (11%)	A single-layer, end-to-side HJ
Shyr et al. [21]	2018	Taiwan, single center	61	2014–N.A	2 (3.3%)	N.A
Guerra et al. [22]	2018	Italy, single center	59	2010–2017	4 (6.8%)	A single-layer, continuous end-to-side HJ
Kim et al. [23]	2017	Korea, single center	51	2015–2017	1 (2.0%)	A single-layer, end-to side HJ
Chen et al. [24]	2015	China, single center	60	2010–2013	5 (8.3%)	A single-layer, end-to side HJ
The present study		Japan, single center	42	2020–2022	1 (2.4%)	A posterior double-layer, interrupted end-to-side HJ

*HJ* hepaticojejunostomy, *N.A.* not available

## Discussion

Over the past several years, various surgical techniques for HJ have been reported in open surgery. To date, there is a lack of randomized controlled trials comparing different HJ techniques. The incidence of biliary complications following HJ in open surgery is relatively low, with bile leakage and anastomotic stenosis rates of up to 8% [3, 8, 25, 26]. However, biliary complications can lead to prolonged hospitalization and increased mortality [3, 4]. Therefore, more attention should be paid to preventing biliary complications. In the setting of minimally invasive surgery, data on surgical techniques and outcomes following robotic HJ are limited [9, 15]. In the present study, we presented a unique surgical technique, posterior double-layer interrupted sutures, in robotic HJ. Our results confirmed the feasibility of this technique for robotic HJ.

Basic principles for the successful implementation of HJ have been reported to be a tension-free reconstruction, well-perfused bile duct and jejunum mucosa, and precise mucosal adaptation between the bile duct and jejunum [8]. Currently, continuous or interrupted suture techniques are commonly used for HJ. In addition, a combination of both the techniques is available. However, the best HJ technique remains debatable. This is because interrupted sutures could have a higher risk of anastomotic leak, whereas continuous sutures are more prone to anastomotic stenosis [9, 27].

**Fig. 2:**
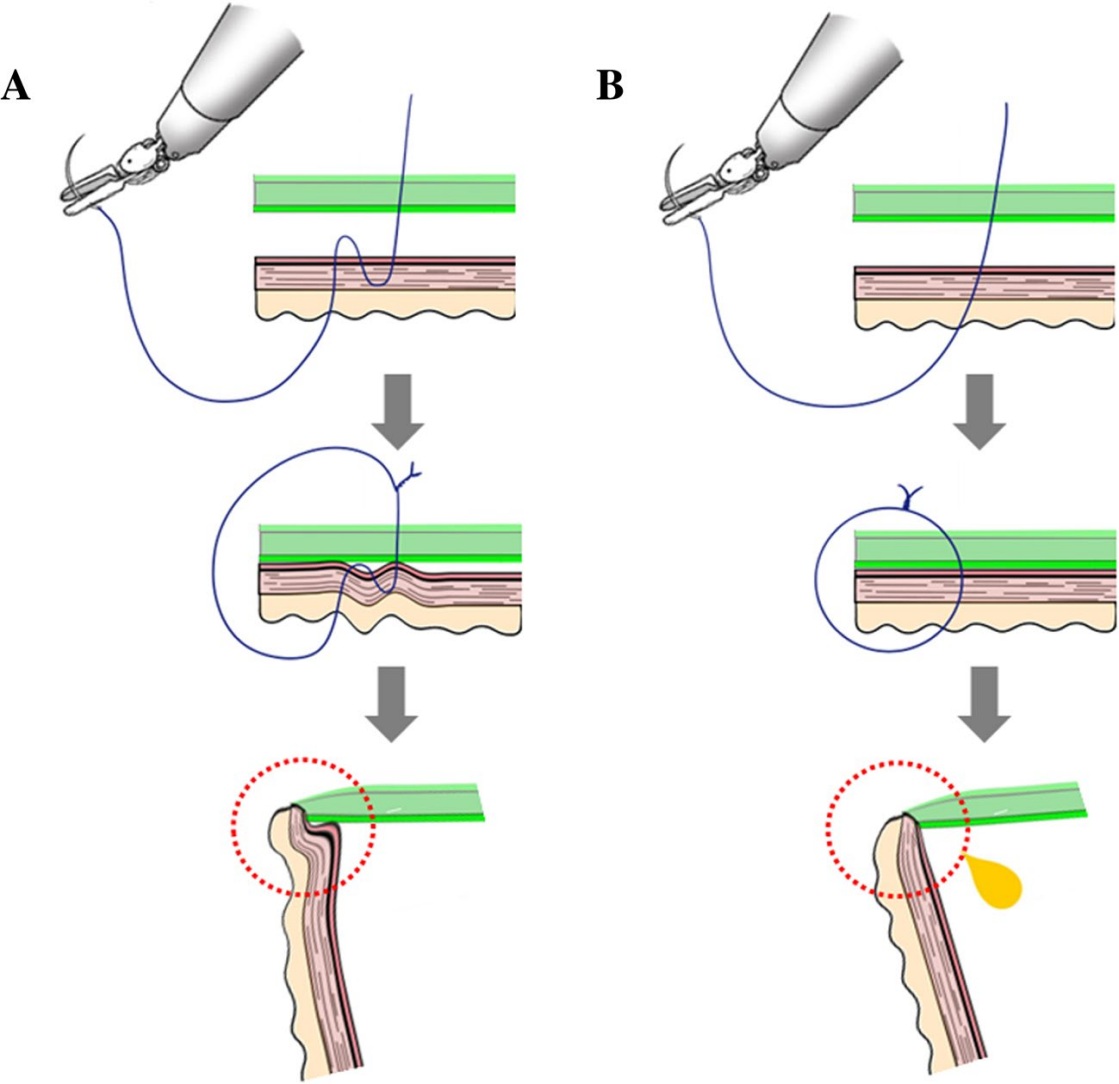
The differences between double- and single-layer hepaticojejunostomy anastomoses. **A** Posterior double-layer interrupted sutures. The bite of the posterior seromuscular layer of the jejunum could cover posterior side of the anastomotic site. Covering the posterior side of the bile duct could prevent minor bile leakage. **B** Single single-layer sutures. The bile duct tearing at the anastomosis could lead to bile leakage

There are several concepts underlying our unique posterior double-layer interrupted sutures. The differences between double- and single-layer HJ anastomoses are illustrated in Fig. 2. The bite of the posterior seromuscular layer of the jejunum could cover the posterior side of the anastomotic site (Fig. 2A). Therefore, covering the posterior side of the bile duct could prevent minor bile leakage, which occurs when the bile duct is torn by a traumatic needle or during ligation with excessive force. Moreover, interrupted sutures could relieve the tension of each suture at the anastomosis. During single-layer suturing, the bile duct tearing at the anastomosis could lead to bile leakage (Fig. 2B).

As bile leakage is more frequent on the posterior side after biliary reconstruction, we believe that this technique could prevent bile leakage, especially on the posterior side. In robotic HJ, bile leakage from the posterior side is invisible and difficult to repair with additional stitches. Therefore, we applied double-layer sutures to the posterior wall. In cases where bile leakage is detected from the anterior side of the HJ, extra stitches can be easily added. Continuous sutures for HJ have been reported to increase the risk of anastomotic stenosis, especially in non-dilated bile ducts [27, 28]. Therefore, our protocol included interrupted sutures for robotic HJ in the standard manner. Regardless of the fact that 74% of the included patients had thin bile ducts of < 10 mm, bile leakage and anastomotic stenosis were found in only one case. Accordingly, posterior double-layer interrupted sutures for robotic HJ could prevent bile leakage and development of anastomotic stenosis.

The standard technique for HJ was found to be a single-layer end-to-side HJ with continuous or interrupted sutures, with the incidence of biliary complications of approximately 5–8% (Table 3). The outcomes of posterior double-layer interrupted sutures for HJ would be relatively better than previous reports using a single-layer technique. These findings could support the feasibility of posterior double-layer interrupted sutures. However, the effectiveness of this unique technique should be validated in other centers.

This study has several limitations. As we applied posterior double-layer interrupted sutures to all HJ anastomoses in the RPD, comparison of this technique to usual single-layer interrupted or running sutures was not possible. This study was designed to confirm the feasibility of our unique technique for robotic HJ with a limited number of patients. Therefore, this technique has not been compared with conventional HJ in open surgery. Future studies should be performed to compare the outcomes of this and conventional HJ techniques. Finally, long-term outcomes of this technique are lacking. Further long-term follow-up studies are required to clarify the significance of this technique.

## Conclusions

The present study demonstrates a unique surgical technique using posterior double-layer interrupted sutures for robotic HJ. Posterior double-layer interrupted sutures may provide acceptable biliary outcomes in robotic HJ. Further investigations regarding long-term outcomes should be performed.

### Supplementary Information

Below is the link to the electronic supplementary material.

Video 1 Robotic hepaticojejunostomy using posterior double-layer interrupted sutures. (MP4 127549 KB)

## Data Availability

All data and materials are available within the paper.
